# Giant Mucinous Carcinoma of the Breast

**DOI:** 10.7759/cureus.3606

**Published:** 2018-11-17

**Authors:** Sara Alothman, Saqib Saeed, Khuram Khan, Alexius Ramcharan, Hector DePaz

**Affiliations:** 1 Surgery, Harlem Hospital Center, New York, USA

**Keywords:** giant, breast cancer, mucinous carcinoma of the breast

## Abstract

We report a case of giant mucinous carcinoma of the breast in a 55-year-old female who presented with left breast lump for two years. The patient did not seek any medical attention for two years. On exam, the lump measured 12 x 14 cm. Core needle biopsy showed invasive mucinous adenocarcinoma without ductal component. The patient underwent neo-adjuvant chemotherapy without any response. She underwent left modified radical mastectomy. All lymph nodes were negative. This was followed by adjuvant chemotherapy. Mucinous carcinoma is a rare type of breast cancer that carries a good prognosis compared to other types of breast cancers. In this report, we presented a giant mucinous carcinoma measuring 14 cm.

## Introduction

Mucinous carcinoma of the breast is a rare type of breast cancer that represents 1–4% of all breast cancers [1]. The natural history of mucinous carcinoma of the breast is not well known. It is characterized by high mucin to epithelium ratio [2]. Mucinous carcinoma carries a favorable prognosis compared to other types of breast cancer and it tends to occur in older patients [3]. We present a case of giant, locally advanced mucinous carcinoma of the breast, non-responsive to neo-adjuvant chemotherapy treated with surgery.

## Case presentation

**Figure 1:**
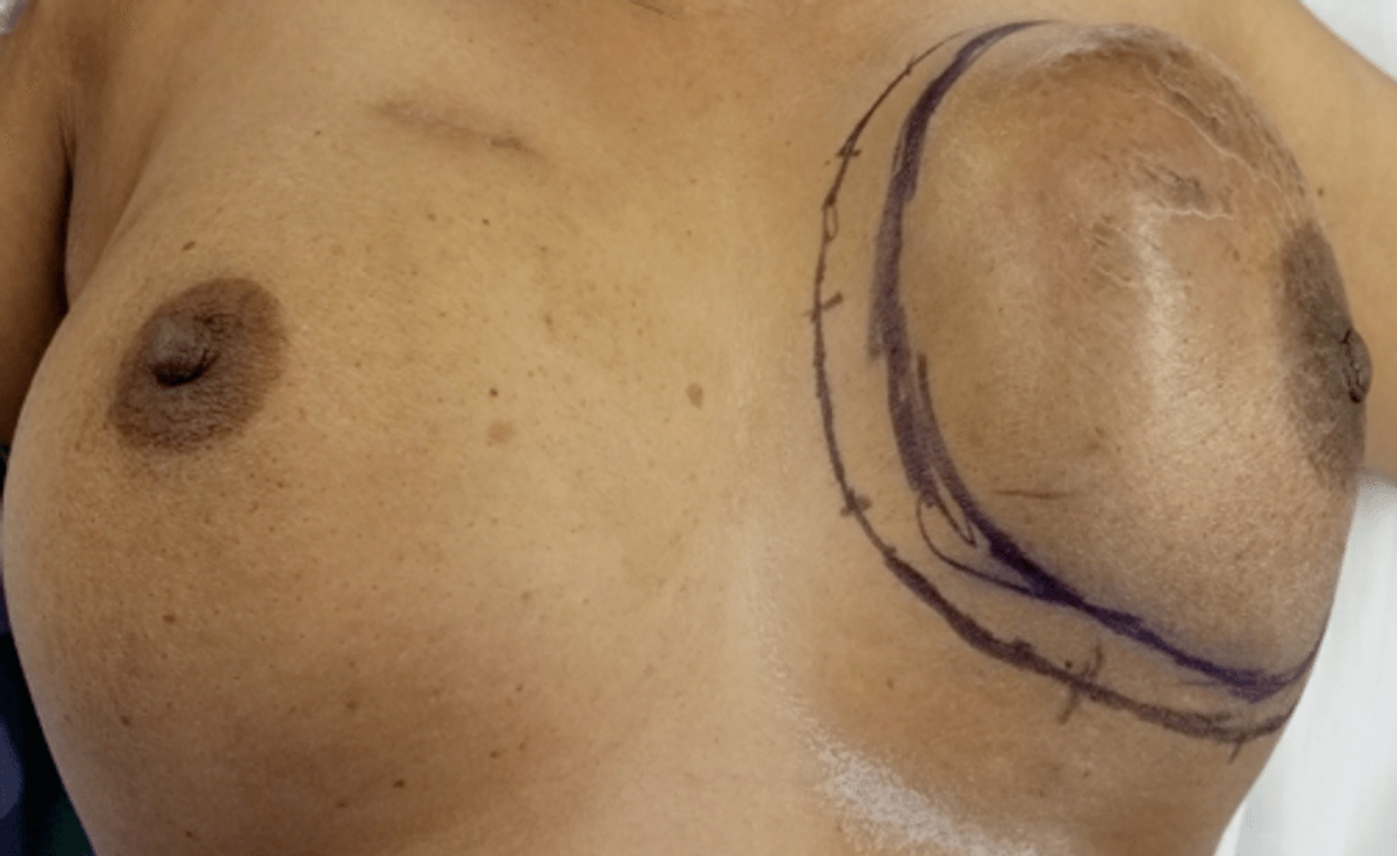
Left breast mass.

**Figure 2:**
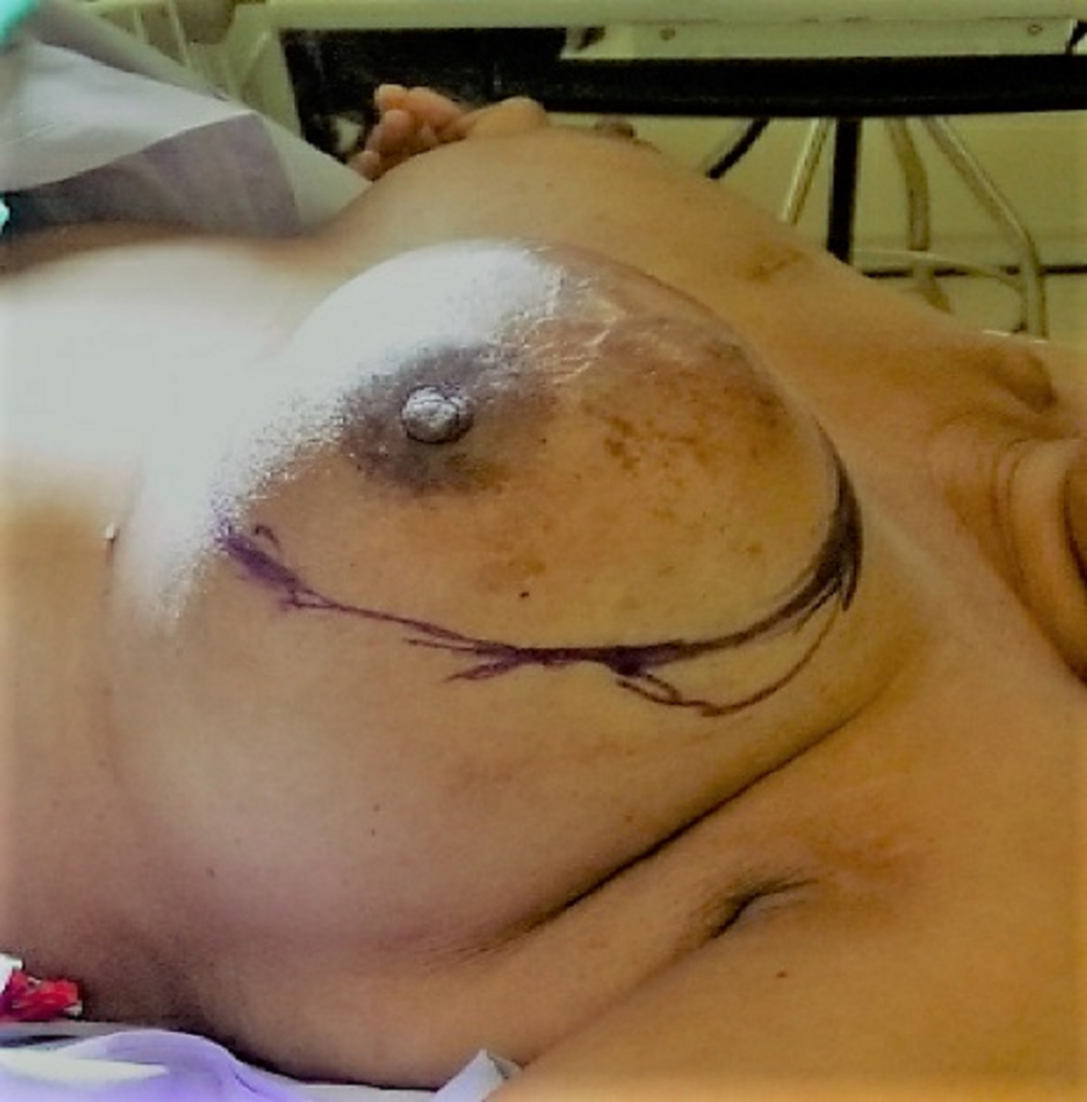
Left breast giant mucinous carcinoma.

A 55-year-old female with no family history of breast cancer presented to breast clinic for evaluation of left breast lump that she noticed two years ago. Initially the lump was painless but recently became symptomatic. Examination revealed a large left breast mass measuring 14 cm x 12 cm involving the medial upper quadrant with overlying skin erythema (Figures 1, 2).

Axillary lymph nodes were not palpable. Right breast exam was within normal limits. Ultrasound confirmed the physical exam findings. Core needle biopsy was performed which came back positive for invasive mucinous carcinoma.

**Figure 3:**
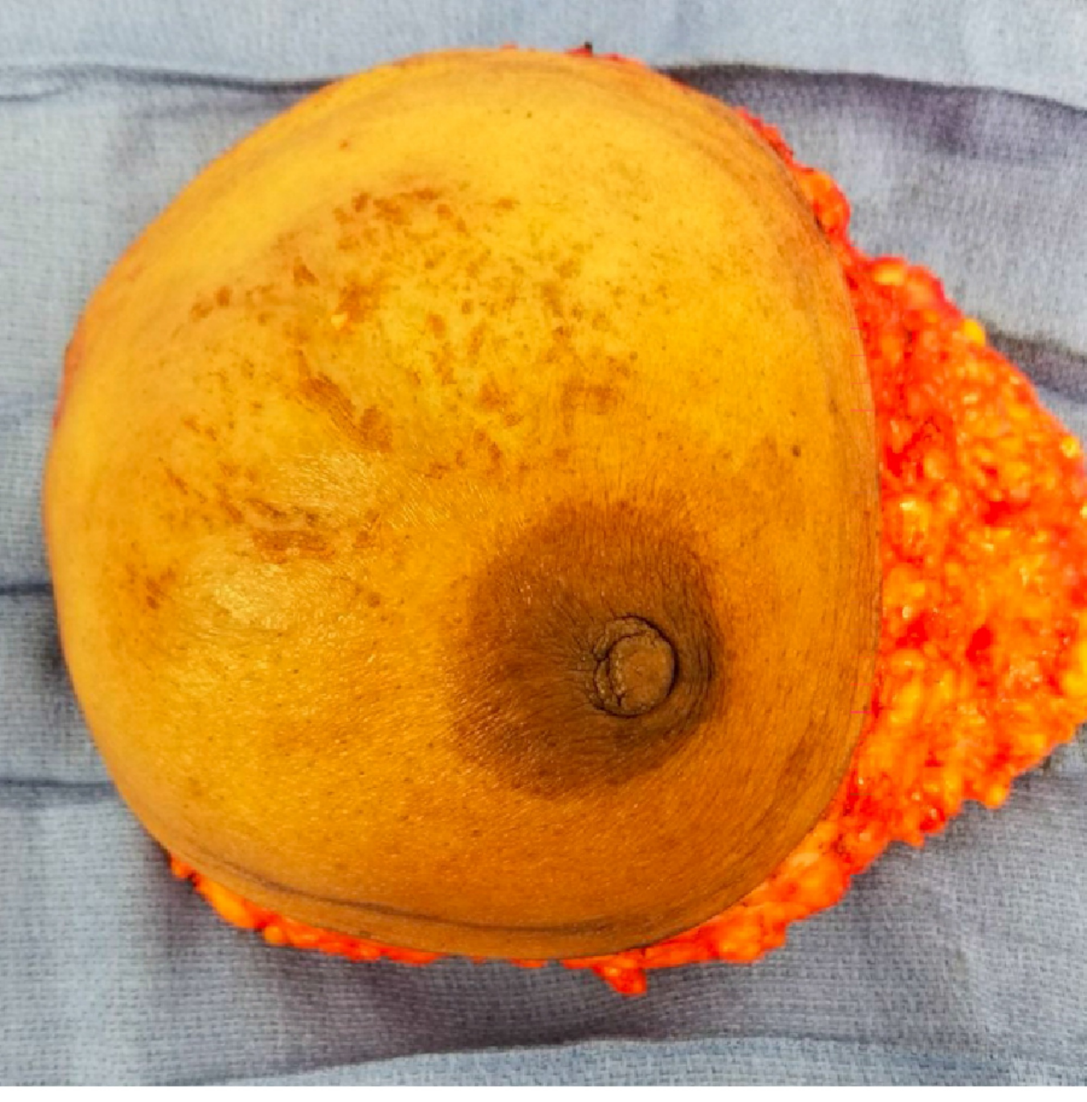
Left breast specimen.

**Figure 4:**
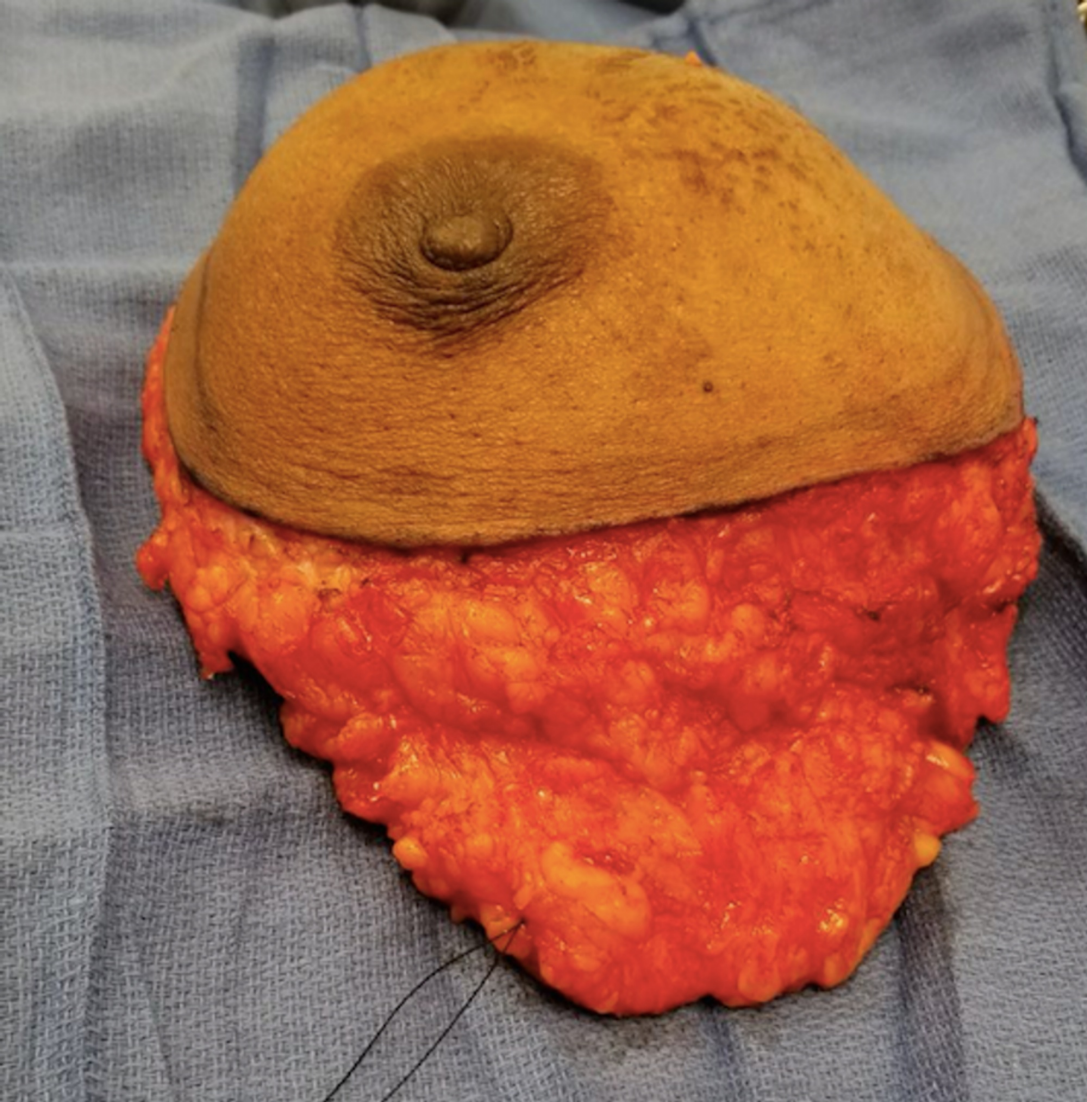
Left breast specimen.

The patient was started on neo-adjuvant chemotherapy. After multiple cycles of chemotherapy, the tumor did not show any regression. With no response to neo-adjuvant chemotherapy, decision was made to proceed with left modified radical mastectomy (Figures 3, 4).

**Figure 5:**
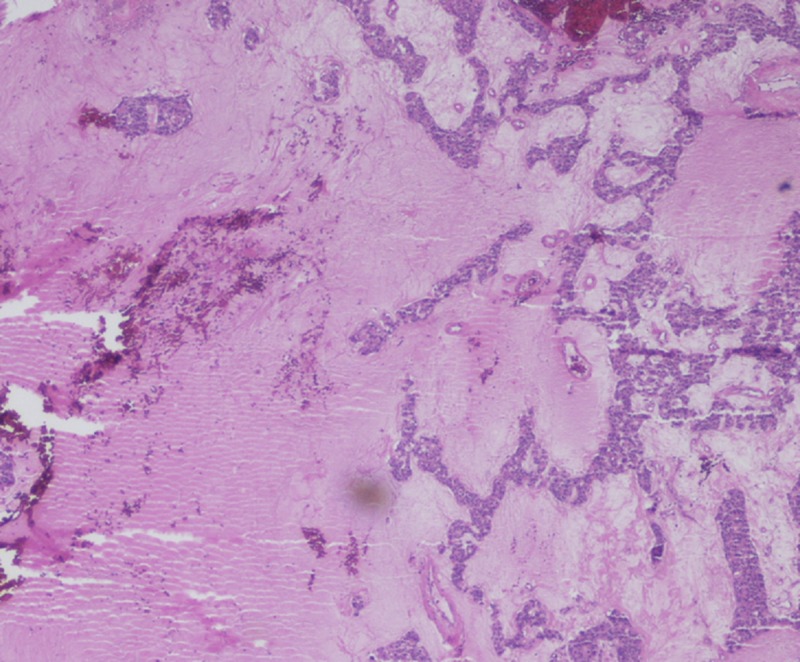
Mucinous breast carcinoma. Clumps of tumor cells lie within pools of mucin, without apparent fibrous reaction.

Surgical pathology showed resected margins to be free of tumor (Figure 5). All the lymph nodes were negative of carcinoma. The tumor was ER/PR positive and HER2/neu negative and was staged T3N0M0. The patient did well after the surgery. She received adjuvant chemotherapy and started on aromatase inhibitor.

## Discussion

The World Health Organization (WHO) defined mucinous carcinoma of the breast as a carcinoma containing a large amount of extracellular epithelial mucus, enough to be visible grossly, and recognizable microscopically surrounding and within tumor cells. It is also named colloid, gelatinous, mucus and mucin adenocarcinoma [3]. It tends to occur at an older age and carries a better prognosis compared to other breast cancer types [4]. It is classified morphologically into two types: pure mucinous carcinoma and mixed mucinous carcinoma, depending on the absence or presence of concomitant areas with typical infiltrating ductal carcinoma. This distinction is important as the pure type has more favorable prognosis than the mixed type [4].

Researchers proposed reasons for favorable prognosis of mucinous breast carcinoma, giving credit to low cellular burden with abundant mucin creating a barrier for the cells from invading the stroma [4].

Patients usually present with a palpable mass but a substantial proportion present with abnormal mammogram with poorly defined or lobulated mass with rare calcifications [5,6]. 20% are mammographically occult [7].

On examination, mucinous carcinomas are generally well-circumscribed and soft. Mucinous carcinomas with high amounts of fibrous stroma are firmer in consistency. A wide range of sizes has been reported in the literature, with an average of approximately 3 cm. Our patient presented with a size of 14 cm.

In our case, the patient had pure mucinous carcinoma. She received neo-adjuvant chemotherapy without clinical response. This is seen in pure mucinous type, where large amount of mucin overestimate the real quantity of tumor cells leading to false interpretation of poor clinical response even though a pathological response is present as presented by Yamaguchi et al. [8]. Therefore, patients with locally advanced tumor are good candidates for primary surgery.

## Conclusions

Pure mucinous carcinoma of the breast carries a favorable prognosis than other types of breast cancer. Lymph node involvement is rare. Neo-adjuvant chemotherapy might fail to shrink the tumor. Primary surgery should be considered as first line therapy in locally advanced cases as clinical response is misleading given high mucin to cell ratio in the tumor.
